# Is having a child with special needs a barrier to physical activity? A mixed design study

**DOI:** 10.1371/journal.pone.0348527

**Published:** 2026-07-13

**Authors:** Melek Kurşunel, Öznur Akpınar, Nazlı Yanar Tunçel, Selahattin Akpınar

**Affiliations:** 1 Department of Sport Science Faculty, Physical Education and Sports Teaching, Karamanoğlu Mehmetbey University, Karaman, Türkiye; 2 Department of Sport Science Faculty, Coaching Education, Karamanoğlu Mehmetbey University, Karaman, Türkiye; 3 Department of Sport Science Faculty, Sports Management, Düzce University, Düzce, Türkiye; Japanese Academy of Health and Practice, JAPAN

## Abstract

Having special needs is considered a difficult situation in society, but perhaps only families experiencing this situation know how challenging it is to be the parent of a child with special needs. Since families plan their entire lives around their children’s needs, they often neglect their own social lives and free time. The aim of this study is to investigate whether having a child with special needs constitutes a barrier to physical activity for parents, using a mixed methods approach. A total of 212 parents caring for individuals with special needs volunteered to participate in the study. The quantitative part of the study used a questionnaire to collect demographic information, as well as scales to measure perceived barriers to physical activity and care burden, and a shortened version of the international physical activity questionnaire. For the qualitative part of the study, a four-question interview form was used. It was observed that, as participants’ scores for physical activity barriers increased, so did their body mass index and care burden scores (p < 0.05). According to the results of the regression analysis, the model of physical activity barriers and care burden status of parents of children with special needs according to the participants’ metabolic equivalent classification was not significant (p > 0.05). However, the results of the qualitative data analysis revealed four barriers that parents indirectly associated with having a child with special needs hindering physical activity: responsibilities, having a child with special needs, psychological state and problems related to sports facilities. These results suggest that quantitative research methods alone may be insufficient for identifying factors hindering physical activity among individuals with special needs. Future studies may wish to conduct applied research projects on physical activity barriers among parents of children with special needs.

## Introduction

The presence of a child with special needs within a family can often lead to frustration and anxiety [1]. All family members may need to adapt their lives to care for and meet the needs of the child [2] Depending on the type of special needs, family members tend to adapt their lives and routines according to the physical, psychological and sociological needs of their children. These changes to daily life can cause various difficulties depending on the care needs of the child with special needs. A qualitative study of families with children with special needs found that the biggest challenges were limitations in their lives due to their children’s special needs and burnout due to caregiving [3]. Caregiving is a multifactorial process involving physical, emotional, social and financial dimensions [4]. The caregiving burden experienced by parents can negatively impact their own and their children’s daily living habits [5]. In particular, parents tend to neglect their own health in order to care for their children [6]. It is well-known that parents’ physical activity levels do not reach the recommended levels, particularly during the time remaining after caring for children during the day [7].

The lifestyles and levels of obesity of parents of young people with special needs have been studied, with 81% of parents found to be overweight or obese [8]. Mothers, who are typically the primary caregivers of children with special needs, face greater challenges in terms of physical inactivity. A cross-sectional study of 69 Turkish mothers of children with motor disabilities found that their physical activity levels were insufficient, with lack of time cited as a barrier [9]. Another issue is how to meet the mobility needs of a child with special needs whose parents are physically inactive. A study of parents of children with special needs in Saudi Arabia found that they supported their children’s participation in physical activity once a week, which was deemed insufficient [10]. Additionally, factors such as the parents’ age and level of education, their own physical activity levels, the type of disability their child has, and the number of children they have were also found to be effective. This study showed that the physical activity of parents of children with special needs directly affects their children. In this context, studies on how to increase parents’ physical activity are becoming increasingly important.

A 14-week family judo programme for parents of children with autism spectrum disorders was found to reduce parental stress and increase daily physical activity levels. Parents in the study also reported increased self-confidence and improved health [11]. Exercise is important for parents of children with special needs, not only for increasing physical activity, but also for its positive effects on factors such as pain, fatigue, stress and sleep quality caused by caregiving. In a Turkish study, mothers with a child with special needs and chronic non-specific low back pain were given breathing exercises to perform over eight weeks. Significant improvements in pain, fatigue, and sleep quality were observed following the intervention [12].

In terms of increasing the participation of children with special needs in physical activity, Shields and Synnot [13] have noted that the burden on parents can be reduced through financial and social support and incentives. However, there is very little research on the barriers to physical activity faced by parents of children with special needs [5,14]. The first of these studies found that parents were not very active, which was linked to health problems. [5]. Another study examined only mothers with and without musculoskeletal pain. It was found that these mothers had low levels of physical activity, which was associated with their quality of life [14]. Although studies on the psychological burden experienced by parents of children with special needs and the stress they face due to the challenges they encounter have been conducted, the relationship between caregiver burden and physical activity levels has only been investigated indirectly [6,15]. A review of the literature also revealed a lack of research into whether having a child with special needs constitutes a barrier to physical activity. These results highlight a significant gap in the literature concerning the potential relationship between parental caregiving burden and physical activity levels and limitations. Additionally, it was noted that studies have used quantitative methods to determine parents’ levels of physical activity and the associated barriers, and that these results may differ when qualitative interviews are conducted. This study will draw clear conclusions by comparing the qualitative and quantitative results regarding the impact of having a child with special needs on parents’ physical activity.

## Materıals and methods

### Research design

This research employs a mixed-methods design, combining quantitative and qualitative approaches. This approach is based on the idea that the outcome of comparing qualitative and quantitative research findings is greater than the sum of its parts [16]. The research design was primarily quantitative, followed by an ‘explanatory sequential’ approach, whereby quantitative data were presented and the results were combined [17]. This research used an explanatory sequential mixed-methods design because most studies on parents of children with special needs tend to be quantitative. However, quantitative data alone may not be sufficient to explain parental experiences. In this context, the qualitative data obtained in this study are necessary for interpreting the quantitative data related to parents’ physical activity limitations. It is extremely important to obtain quantitative data first and then ask qualitative questions related to the quantitative data in order to interpret the findings. This reveals the underlying reasons and transparency of the findings.

### Research group

Purposive sampling was used to select the study sample. It was decided that 200 participants meeting the appropriate criteria would be sufficient [18]. A total of 212 people (181 female, 31 male) living with special needs in Türkiye, voluntarily participated in the study. Quantitative data was collected in person between June 15 and September 15, 2024, after obtaining the participants’ verbal consent in the presence of a witness. Subsequently, appointments were arranged with volunteers for qualitative interviews, and the interviews were conducted. Qualitative interviews were recorded with the participants’ verbal consent. Personal information such as names or surnames was not included during the interviews. Participants were adults (18 years of age or older). The mean age of the participants was 40.5 years (sd:10.6), the mean height was 163.1 cm (sd: 8.0), the mean body weight was 70.6 kg (sd:14.1) and the mean BMI was 26.5 kg/m² (sd:5.0). The other demographic characteristics of the participants are presented below (Table 1).

**Table 1:** Demographic characteristics of participants.

	Group	F	%
**Marital status**	***Married***	184	86.8
	***Widow***	28	13.2
**MET classification**	***Inactive***	70	33.0
	***Active***	59	27.8
	***Very Active***	83	39.2
**Educational level**	***Primary school***	63	29.7
	***Secondary school***	41	19.3
	***Tertiary school***	82	38.7
	***University***	26	21.2
**Occupation**	***Housewife***	147	69.3
	***Officer***	25	11.8
	***Self-employment***	33	15.6
	***Retired***	7	3.3
**Income level**	***17.000 and low***	105	49.5
	***17.000-25.000***	36	17.0
	***25.000-35.000***	22	10.4
	***35.000 and high***	49	23.1
**Chronic disease**	***Have***	60	28.3
	***Dont have***	152	71.7
**Having special needs**	***Yes***	13	6.1
	***No***	199	93.9
**Children’s special need type**	***Orthopedic***	36	17.0
	***Vision***	3	1.4
	***Hearing***	3	1.4
	***Speech and Language***	67	31.6
	***Chronic Illness***	2	0.9
	***Mental***	69	32.5
	***Multiple***	32	15.1

### Data collection instruments

The researchers developed a questionnaire that included questions about the participants’ gender, age, height, weight, number of children, level of education, marital status, income, whether they had any special needs, the special needs category of their child, and whether they received social support.

#### Demographic information questionnaire.

The Perceived Barriers to Participation in Physical Activity Scale, developed in 2006, was used to determine perceived barriers to physical activity [19]. A validity and reliability study of the scale for the Turkish population was conducted in 2023 [20]. The scale consists of 17 items and four sub-dimensions, with scores ranging from 0 (low) to 10 (high). The sub-dimensions of body image and psychosocial anxiety (items 3, 6, 10, 13 and 16), fatigue and laziness (items 1, 2, 5, 8, 9 and 12), obligations and time constraints (items 4, 7 and 11) and environment and facilities (items 14, 15 and 17) are calculated as a sum of the scores for each item. The total Cronbach’s alpha internal consistency coefficient of the scale is 0.83. In this study, the overall Cronbach’s alpha coefficient was 0.86, with sub-dimension coefficients of 0.87 for body image and psychosocial anxiety, 0.72 for fatigue and laziness, 0.77 for obligations and time constraints, and 0.63 for environment and facilities.

#### Perceived barriers to participation in physical activity scale.

The Caregiver Burden Scale was developed to measure the stress experienced by caregivers of older people in need of care [21]. Consisting of 22 items on a 4-point Likert scale (never (1), rarely (2), sometimes (3), often (4)), the scale has 5 sub-dimensions. A study to adapt the original scale for use in Turkish culture was conducted in 2014 [22]. The sub-dimensions of the scale were calculated by summing the items and dividing by the number of items: general strain (items 1, 3, 4, 5, 5, 7, 10, 14, 19); isolation (items 8, 12, 22); frustration (items 2, 13, 18, 20, 21); emotional involvement (items 6, 11, 16); and environment (items 9, 15, 17). The overall Cronbach’s alpha coefficient for the scale’s internal consistency was found to be 0.91, with sub-dimensions of general distress (0.87), isolation (0.70), frustration (0.76), emotional involvement (0.70), and environment (0.53). In a subsequent study, the overall Cronbach’s alpha coefficient for the scale was 0.90, with sub-dimensions identified as general distress (0.82), isolation (0.59), frustration (0.67), emotional involvement (0.76), and environment (0.63).

#### Caregiver burden scale.

Participants’ levels of physical activity were assessed using the International Physical Activity Questionnaire (IPAQ)-Short Form. The original version of the questionnaire consists of 27 questions and was developed by Michael Booth [23]. It was adapted for use in a Turkish context in 2005 [24]. Consisting of seven questions, the scale includes enquiries about sitting, walking, and moderate- and vigorous-intensity activities undertaken in the past week. Individuals’ MET levels are calculated by answering the questions in days and minutes. One MET corresponds to the metabolic rate of the body at rest. MET levels (MET minutes per week) are calculated by multiplying the day and minute values obtained from the scale. Sitting is multiplied by 1.5, walking by 3.3, moderate-intensity activities by 4.0, and high-intensity activities by 8.0. The resulting values are interpreted as follows: inactive (≤600 MET minutes/week), active (600–3,000 MET minutes/week) and very active (>3,000 MET minutes/week) [25].

#### International physical activity questionnaires-short form.

The qualitative phase of the research was conducted with the participants who participated in the quantitative part of the research and those who wanted to participate in the qualitative interview. The “data saturation point” was used as a criterion to determine the number of participants [26]. The saturation point is defined as “similar responses in the interviews conducted after 10 interviews” [27]. In this context, 20 (female: 14, male: 6) parents voluntarily participated in the qualitative interview.

#### Qualitative research group.

The researchers prepared an interview form consisting of four questions. When designing the interview form, the sub-dimensions of the scales used in the quantitative part of the research were taken into account. Expert opinion was sought to ensure the content and face validity of the form. Following this, the final version of the interview form was prepared. The questions are:

#### Qualitative interview form.

1. How do your daily responsibilities affect your level of physical activity?
2. How do the sports facilities in your neighbourhood affect your level of physical activity?
3. How does your emotional state affect your level of physical activity?
4. How does living differently to other parents affect your level of physical activity?

### Data analysis

First, the kurtosis and skewness values of the data obtained from the participants were analysed to determine whether the data were normally distributed. The participants had the following values: BMI (skewness: 0.62, kurtosis: 0.53); MET score (skewness: 0.96, kurtosis: −0.40); physical activity barrier score (skewness: 0.37, kurtosis: −0.31); and care burden score (skewness: 0.33, kurtosis: −0.47). The results of the kurtosis and skewness analysis showed that the data fell within the ± 1.5 range, indicating a normal distribution [28]. Following this, Pearson’s correlation analysis was used for relational comparisons, while multinomial logistic regression analysis was used to determine the relationship between categorical variables. All quantitative analyses were performed using the Jamovi statistical programme (version 2.3.28.0). A 95% confidence interval and a 0.05 significance level were used. Qualitative data were analysed thematically using an inductive, constant comparative approach [29]. Following Braun and Clarke’s [29] six-step thematic approach, the principal investigator and another researcher examined each qualitative interview in detail and created codes. The researchers then categorised these codes according to their similarities and compared the results to finalise the themes. The principles of sequential mixed-methods research design were then applied to interpret the quantitative and qualitative results comprehensively [30].

#### Ethical approval.

This study was conducted in accordance with the principles of the Declaration of Helsinki. Ethical approval for the study was granted by the Scientific Research and Publication Ethics Committee of the Faculty of Social and Human Sciences at Karamanoğlu Mehmetbey University (Approval number; 08–2024/191, Approval date 21/05/2024). Informed consent in writing was obtained from all participants.

## Results

This study was conducted to predict whether having a child with special needs poses a barrier to physical activity. The following results were obtained. First, the quantitative results of the study were analysed and presented (Table 2).

**Table 2:** The mean scores of the participants MET, Physical activity barriers and care burden.

Variables	n	x̄	sd
MET	212	1668.5	1542.1
Perceived Barriers to Participation in Physical Activity Scale	212	3.7	2.1
Caregiver Burden Scale	212	2.2	0.6
***Physical Activity Barriers Scale Sub-Dimensions***			
Body image and psychosocial anxiety	212	2.1	2.3
Fatigue and laziness	212	4.0	2.4
Obligations and time constraints	212	5.2	3.1
Environment and facilities	212	2.7	2.5
***Care Burden Scale Sub-Dimensions***			
General distress	212	2.7	0.7
Isolation	212	2.2	0.9
Frustration	212	2.3	0.8
Emotional involvement	212	1.8	0.8
Environment	212	2.0	0.8

Sd: Standart deviation.

### Quantitative results of the study

The table below (Table 3) presents the results of the pairwise comparisons of the mean values for BMI, MET, physical activity barriers and care burden of the participants.

**Table 3:** Participants’ BMI, MET, physical activity barriers and care burden correlation test results.

		BMI	MET	Physical Activity Barriers
**MET**	r	−0.15*		
	p	0.03	---	
**Physical Activity Barriers**	r	0.15*	0.06	
	p	0.03	0.4	---
**Care Burden**	r	−0.02	−0.03	0.15*
	p	0.8	0.6	0.04

**Fig 1:**
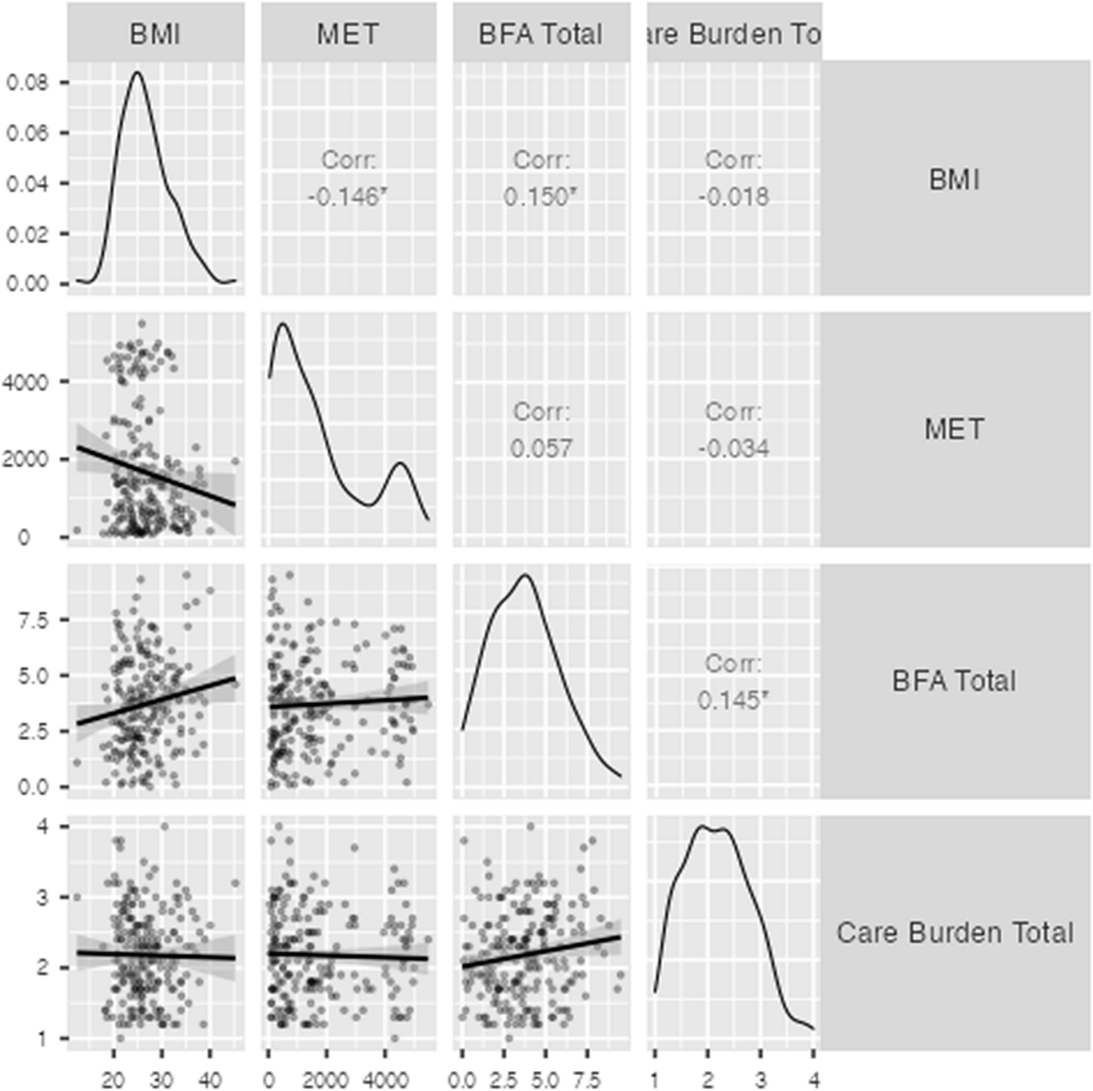
The results of the correlation analysis of the participants.

As can be seen in Table 3, there was a weak negative correlation between participants’ BMI and MET scores (r = −0.15, p < 0.05). Similarly, there was a low positive correlation between participants’ scores for barriers to physical activity and their BMI (r = 0.15, p < 0.05). A small positive correlation was observed between caregiver burden scores and physical activity barriers scores (r = 0.15, p < 0.05). Fig 1 shows the results of the correlation analysis. The results of the regression analysis of participants’ BMI, MET, caregiving burden, and physical activity barrier scores are presented in Table 4 below.

**Table 4:** The results of multinomial logistic regression analysis according to the MET classification of the participants.

	Predictor	Estimate	S.E.	Z	C.I.	O.R.	p
**Active – Inactive**	İntercept	0.003	0.69	0.00	0.26-3.86	1.00	0.99
	PA barriers	0.005	0.09	0.06	0.85-1.19	1.01	0.95
	Care burden	−0.089	0.29	−0.31	0.52-1.60	0.92	0.76
**Very Active -Inactive**	İntercept	0.511	0.63	0.81	0.49-5.72	1.67	0.42
	PA barriers	0.035	0.08	0.43	0.89-1.21	1.04	0.67
	Care burden	−0.209	0.26	−0.79	0.48-1.36	0.81	0.43
N:212, R^2^ = 0.02 (Negelkerkes) Model = *x*^*2*^(2)=0.77, p = 0.94							
*PA: Physical activity, S.E: Standart error, C.I: Confidence interval, O.R: Odss ratio*							

**Fig 2:**
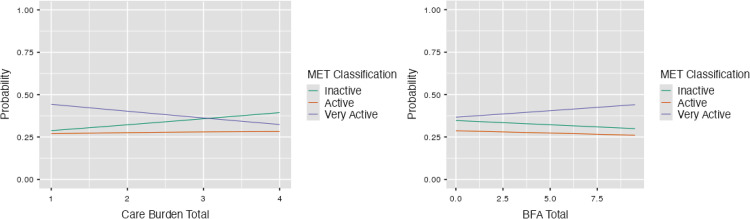
The results of the regression analysis of the participants.

Examining Table 4, it can be seen that the model of physical activity barriers and care burden status of parents of children with special needs, according to the MET classification of participants, is not significant (R² = 0.02, p > 0.05). Considering the independent variables, it can be seen that being active is likely to increase physical activity disability scores by 1.01 times and care burden scores by 0.9 times compared to being inactive; however, this probability is not statistically significant (p > 0.05). Being very active was 1.04 times more likely to increase physical activity disability scores and 0.8 times more likely to increase care burden scores than being inactive; however, this probability was not statistically significant (p > 0.05). Fig 2 shows the results of the regression analysis.

The results of the quantitative analysis were examined. The qualitative analysis of the data, which is another stage in the research design process, is presented in Table 5.

**Table 5:** Thematic and sub-themes that reflect the participants’ views on the perceived physical activity barriers.

Themes (N:4)	Sub-Themes (N:9)	Example quotes
Responsibilites	Lack of time (n:8)	*‘I can’t do any physical activity during the day because I have responsibilities. I have to run after and take care of my child.’* *We don’t have time to play sports because we have to look after the children during the day. At weekends and during the week, we can never do it when they are normally at home.“*
	Workload (n:6)	*‘I work, and unfortunately I don’t have time for sports as I have to look after my children the rest of the time.’* *I am currently working as a civil servant, so my working hours are from 8 am to 5 pm. Therefore, it is not possible for us to set aside time for physical activity.“*
Having disabilities child	Putting the child first (n:6)	*‘You cannot leave your child at home alone or go out by yourself. Either you can leave a healthy child at home and go out, or you can go out yourself. But we can’t do that, so how can we play sports or take part in physical activities?’* *‘If you have a typical child, you can tell them to go and play by themselves, but if you have a child with special needs, you have to spend time with them. They can’t do anything without us.“*
	Behaviors of exclusionary (n:3)	*‘I feel ostracized by society as a whole, including my own family and relatives. They have normal, healthy children and grandchildren, yet we are ostracised, which hurts us.* *When we take our children out for a walk, people coming from the opposite direction are disturbed by my child’s movements. The looks of other people affect the children, so of course, we don’t take walks with my child anymore.“*
Psycgological state	Stress (n:3)	*“To play sports, you need a carefree, peaceful and happy life without stress. Since I don’t have a comfortable life and experience stress due to my daily responsibilities, I don’t have the time or energy to play sports.“* *I experience stress because of the responsibilities associated with my daily life. During these stressful periods, I try to relax by doing sports, but I can’t do it all the time.“*
	Lack of motivation (n:4)	*‘’When I’m feeling down, I don’t want to move at all. I always want to go to bed because I am sad about the situation our children are in.“* *If the children are happy during the day, I’m happy too. But when I feel overwhelmed, I want to go out, even though I can’t leave the children alone. When that happens, I don’t feel like moving.“*
Sport facilities problems	Insufficient facility (n:4)	*‘’There are no sports fields or walking areas around here. If there were, I would go for a walk.”* *‘At the moment, there is nowhere near my house where I can play sports, but it would be great if there was. I could just put on a tracksuit and go to the sports field to do all kinds of activities and sports.“*
	Expensive facility (n:1)	*‘’The gym prices are already very high. We don’t have the financial means to afford a gym membership.”*

### Qualitative results of the study

#### Responsibilites.

The most common barrier to physical activity reported by participants was a lack of time. They said that their responsibilities at home and caring for more than one child with special needs meant they did not have time for physical activity. They explained this as follows:

*‘As I have two children with special needs, they have a number of daily needs. Unfortunately, we neglect our own physical activities because we spend more time with them to meet their needs.“*

*‘I have three children with special needs and my husband has died. I take care of them all day; I have no time for sport or physical activity.“*

They also reported that working parents are tired when they get home from work and have no time for physical activity. He emphasised the following:

*‘After working in the evening and feeling tired from the day, I have no time for physical activity.’*

#### Having disabilities child.

The second most commonly reported barrier to physical activity was ‘having a child with special needs’. Participants stated that they did not consider physical activity because they prioritised their child/children’s needs. They explained as follows:

*“We don’t have a normal life because we have children who need to be taken care of like babies, even though they are adults. We have adult children whom we raise as if they were babies. The effort we put into them is more tiring. Therefore, probably because of this situation, we cannot do sports or physical activities.”*

*‘’So, when you have a child with special needs, a mother’s time is always taken up by them. I used to live with my child, who went to school from morning until noon, leaving me with some free time. Now he is always at home, and because of my responsibilities, I cannot do sports in my spare time as I cannot leave my child with special needs with someone else.’*

They also regretted that both children and parents were disturbed by the exclusionary behaviours and looks of people around them in the community when they wanted to take part in sports and physical activities. For example:

*“I used to go swimming and take my child with me, which was good for him. However, due to prejudices against people with special needs in society, I cannot take my child to such environments. This situation therefore has a negative effect on our physical activity.”*

*‘’When people are not emotionally well, they don’t want to do sports or even eat, so it affects my physical activity.“*

#### Psycgological state.

The third most common barrier to physical activity, according to participants, was their state of mind during the day. Most participants said that they felt unhappy and stressed, and that these feelings prevented them from being active. They expressed this as follows:

*‘I’m always emotional during the day. I miss laughing; I laugh, but not like that, and I envy those who can. I don’t even think about physical activity or sport because I find it hard emotionally“.*

Participants also stated that their motivation decreased because of the negative emotions they experienced. One participant explained it like this:

*“For the last year, whenever I’ve felt emotionally down, I’ve locked myself in the house. I used to go out for a 40-minute walk and feel motivated. Now, on the contrary, I stay at home and go to bed, and I don’t know what else to do”.*

### Sports facilities problems

The final barrier to physical activity that was most frequently mentioned by participants was ‘problems with sports facilities’. Participants said that having a sports facility near their home would encourage them to be physically active, but that there were still not enough sports parks. They said the following:

*“It would be good to have walking areas around my house. My daughter and I are both overweight. When we go to the park, if there is sports equipment, I try to do some sports.”*

*‘I benefit from the playing fields around my house, but they are not enough. There is sports equipment in the city parks. We go there and try to do activities together, but it is not enough.“*

## Discussion

This is the first study to use a mixed-methods approach to investigate whether having a child with special needs hinders parents’ physical activity. Establishing whether this is the case could inform solutions aimed at increasing parents’ levels of physical activity. The quantitative results of the current study showed that, as the care burden and barriers to physical activity increased, so did BMI. However, the model of barriers to physical activity and care burden status of parents of children with special needs according to the MET classification was not significant. These results were not confirmed by the qualitative findings, which revealed that barriers to physical activity arising from having a child with special needs were a key theme. Examining the content of responsibilities arising from having a child with special needs, having a special child, emotional state, and problems with sports facilities revealed that parents associated these barriers with having a child with special needs. Therefore, it can be concluded that studies in the literature [4,31–35] that reveal only quantitative data on physical activity barriers for parents with children with special needs are insufficient.

In the quantitative phase of this study, our results showed that the BMI of parents with children with special needs increased as the level of care burden and barriers to physical activity increased. It is well-known that mothers with children with special needs are less physically active than mothers with healthy children [31]. Having a child with special needs can lead not only to physical inactivity, but also to a care burden. This study investigated the relationship between physical activity and care burden among parents of children with special needs in Türkiye. It was found that most parents had a low level of physical activity, and that most of them experienced a mild to moderate care burden. Furthermore, it was found that parents with high levels of physical activity experienced a higher care burden [4]. However, in contrast to this finding, it was observed that during the Covid-19 pandemic, mothers of children with autism spectrum disorder experienced higher levels of psychological distress than mothers of healthy children, though this was not associated with physical activity levels. Additionally, a positive correlation was observed between maternal happiness and physical activity levels [36]. These findings suggest that the relationship between caregiving burden and physical activity levels among parents of children with special needs may vary, as may the probability estimates for the future. In this context, the lack of significance of the probability estimation results for the model of barriers to physical activity and the care burden status of parents with children with special needs according to the MET classification in this study supports the differences observed in previous studies. The lack of correlation and probability in these quantitative findings also highlights the need for qualitative data analysis.

In the qualitative phase of this study, we found that parents’ perceptions of barriers to physical activity were related to responsibilities (e.g., lack of time and work commitments), having a child with special needs (e.g., putting the child first and social exclusion), psychological state (e.g., stress and lack of motivation), and problems with sports facilities (e.g., inadequate and expensive facilities). A systematic review was conducted on the perspectives of parents of young people with disabilities regarding physical activity. According to the results, families identified factors such as a lack of time and programmes for children with disabilities, as well as the nature of the child’s disability, as barriers to physical activity [37]. However, a study investigating the physical activity levels of children with special educational needs and their families in Türkiye found no significant difference in physical activity levels according to the type of disability [34]. Notably, when examining the types of disabilities of children with special needs in this study, the majority (32.5%) were classified as having intellectual disabilities. Societal perceptions of fear surrounding people with intellectual disabilities may prevent parents from comfortably taking their children outdoors, engaging them in social activities and sports. A systematic review investigating the physical activity levels of parents of children with autism spectrum disorders found that parents are more likely to exercise if they can bring their children with them [32]. This research demonstrates that social exclusion affects parents of children with special needs.

The fact that these parents are aware of this social exclusion may also create fears for the future. In one study, families with special needs stated that their main concern was being left alone if they died before their children [3]. This indicates that parents with children with special needs prioritise their children. A variety of factors need to be considered to prevent social exclusion and increase the physical activity levels of children with special needs and their parents. A study investigating the experiences of parents of children with intellectual disabilities in increasing physical activity identified four themes: social support, family fears, family responsibilities and involving the child in sports or games [38]. This finding raises questions about the importance of social support and practices that increase physical activity, as well as how these practices should be designed. The study examined factors that facilitate or hinder regular physical activity as reported by parents of young people with neurodevelopmental and physical conditions. The study found that physical activity should be fun for all family members, siblings and parents should be encouraged to participate, and participation should be ensured for low-income families [33]. These findings all support the perceptions of barriers to physical activity held by parents of children with special needs in this study.

It is worth noting that 85% of the parents in the current study were mothers. It is well-known that mothers dedicate a lot of energy to caring for their children and organising their participation in daily life [39]. This situation also highlights the importance of maternal health for both mother and child, given that mothers are typically the primary caregivers for children with special needs [40]. Mothers with children with special needs experience more psychological distress and anxiety than parents with healthy children [41,42], because they are the primary caregivers both at home and in the care of the child. A study of mothers of children with special needs in Türkiye found that the majority had low levels of physical activity. Furthermore, no significant differences were found in the caregiving burden, self-efficacy, or depression scores of these mothers according to their physical activity levels [35]. In light of this information, it is believed that improving the health of mothers in all areas will benefit children with special needs. For instance, the Healthy Mothers Healthy Families programme for mothers of children with special needs resulted in improvements in mental health, reduced stress and anxiety levels, increased self-awareness, and greater participation in leisure activities with their children [43]. Additionally, the fact that sample groups of parents with special needs in the literature consist only of mothers is a significant shortcoming, meaning there is little information about fathers with children with special needs.

The results of this study show that barriers to physical activity among parents of children with special needs are associated with having a child with special needs. It is noteworthy that mothers are the primary caregivers of children with special needs. During the data collection process, it was observed that the majority of people accompanying children to special educational institutions were mothers. Qualitative interviews with parents revealed that the biggest perceived barrier to physical activity is lack of time due to daily childcare and work responsibilities. In addition to the responsibility of having a child with special needs, society’s attitude towards these needs can also reduce parents’ motivation and tendency to be physically active. This situation affects not only the psychological state and level of physical activity of parents, but also whether children with special needs do sports or physical activity outside. For this reason, parents prefer to take their children to outdoor walking areas and sports parks close to their homes, rather than to sports facilities open to all. One study showed that parents of children with special needs who had made walking a lifelong habit were more likely to be physically active [32]. Perhaps this is because parents of children with special needs.

## Conclusion

This study shows that barriers to physical activity among parents of children with special needs are related to the experience of having a child with special needs. The results of this study are of interest to various disciplines, including sports science, special education, public health, psychology, local government and health policy. Notably, society’s perception of people with special needs is found to cause many cascading factors. Additionally, the lack of sports facilities for children with special needs and their families, or the fact that sports facilities do not aim to integrate individuals with and without special needs, has emerged as a major deficiency. This study and others in the literature have shown that mothers bear a significant burden when caring for children with special needs. In this context, the physical and mental health of mothers is of great importance when caring for these children.

### Limitations

This study employed quantitative and qualitative research methods to investigate whether having a child with special needs hinders physical activity. It did not address the impact of laws and regulations on people with special needs other than the children of parents. Additionally, no qualitative research was conducted to determine why sports facilities do not cater for people with special needs. These two factors can be considered limitations of the study.

### Solutions

The results of this study showed that the physical activity of parents with children with special needs was negatively affected by their children’s specific requirements. Additionally, the fact that the majority of the care burden falls on mothers was found to negatively impact their physical and psychological health. In this context, there is a need for sports facilities to be built and supported by laws and regulations, and for local governments to implement practical projects to increase the physical activity levels of parents with children with special needs.
