# Supplementary material for: Is having a child with special needs a barrier to physical activity? A mixed design study
Source: PLoS One. 2026 Jul 13;21(7):e0348527. doi: 10.1371/journal.pone.0348527 (PMC13362118; doi:10.1371/journal.pone.0348527)
Supplement: Attachment — Submitted filename: Response to Reviewers.docx [file pone.0348527.s002.docx]

**Dear Editor:** The researchers' names have been updated. Due to her divorce, Melek GÜLER's surname has been changed to Melek KURŞUNEL. Her email address has also been updated. The names of the other researchers have not changed (lines: 8 and 35). Information regarding ethical approval decisions has been added to the Methods section (lines: 245–249). Tables 1, 2 and 5 are referenced in the text (lines: 160, 255 and 295). Figures 2 and 3 are referenced in the text (lines: 273 and 29).

**Dear Reviewer 1:** Reference sources have been added to support some of the statements in the introduction (lines: 80–83). The final paragraph of the introduction explains why this research was necessary and discusses the research gap in the literature. References have also been added (lines: 112–122). The rationale for using a mixed-methods design is explained in detail in the Methods section (lines: 136–144). The following sources have been added to the references section.( lines: 510, 513, 516, 538 and 542).

**Dear Reviewer 2**: When discussing mixed methods in research design, the work of Creswell and Clark (2007) is referenced. However, a research design diagram has been added to improve understanding of the design (line: 146).
