# Supplementary material for: Is having a child with special needs a barrier to physical activity? A mixed design study
Source: PLoS One. 2026 Jul 13;21(7):e0348527. doi: 10.1371/journal.pone.0348527 (PMC13362118; doi:10.1371/journal.pone.0348527)
Supplement: Peer review history

# Peer review: Decision Letter 0

**DOI:** 10.1371/journal.pone.0348527.r001

**Authors:** Hidetaka Hamasaki

16 Mar 2026

PONE-D-25-53816

Is having a child with special needs a barrier to physical activity? A mixed design study

PLOS One

Dear Dr. KURŞUNEL,

Thank you for submitting your manuscript to PLOS ONE. After careful consideration, we feel that it has merit but does not fully meet PLOS ONE’s publication criteria as it currently stands. Therefore, we invite you to submit a revised version of the manuscript that addresses the points raised during the review process.

Please submit your revised manuscript by Apr 30 2026 11:59PM. If you will need more time than this to complete your revisions, please reply to this message or contact the journal office at plosone@plos.org. When you're ready to submit your revision, log on to https://www.editorialmanager.com/pone/ and select the 'Submissions Needing Revision' folder to locate your manuscript file.

Please include the following items when submitting your revised manuscript:

- A letter that responds to each point raised by the academic editor and reviewer(s). You should upload this letter as a separate file labeled 'Response to Reviewers'.
- A marked-up copy of your manuscript that highlights changes made to the original version. You should upload this as a separate file labeled 'Revised Manuscript with Track Changes'.
- An unmarked version of your revised paper without tracked changes. You should upload this as a separate file labeled 'Manuscript'.

-->If you would like to make changes to your financial disclosure, please include your updated statement in your cover letter. Guidelines for resubmitting your figure files are available below the reviewer comments at the end of this letter.

If applicable, we recommend that you deposit your laboratory protocols in protocols.io to enhance the reproducibility of your results. Protocols.io assigns your protocol its own identifier (DOI) so that it can be cited independently in the future. For instructions see: https://journals.plos.org/plosone/s/submission-guidelines#loc-laboratory-protocols. Additionally, PLOS ONE offers an option for publishing peer-reviewed Lab Protocol articles, which describe protocols hosted on protocols.io. Read more information on sharing protocols at https://plos.org/protocols?utm_medium=editorial-email&utm_source=authorletters&utm_campaign=protocols.

We look forward to receiving your revised manuscript.

Kind regards,

Hidetaka Hamasaki

Academic Editor

PLOS One

Journal Requirements:

When submitting your revision, we need you to address these additional requirements.

1. Please ensure that your manuscript meets PLOS ONE's style requirements, including those for file naming. The PLOS ONE style templates can be found at

https://journals.plos.org/plosone/s/file?id=wjVg/PLOSOne_formatting_sample_main_body.pdf and

https://journals.plos.org/plosone/s/file?id=ba62/PLOSOne_formatting_sample_title_authors_affiliations.pdf

2. Please include your full ethics statement in the ‘Methods’ section of your manuscript file. In your statement, please include the full name of the IRB or ethics committee who approved or waived your study, as well as whether or not you obtained informed written or verbal consent. If consent was waived for your study, please include this information in your statement as well.

3. In the online submission form, you indicated that this text is appropriate if the data are owned by a third party and authors do not have permission to share the data. ButThe article includes some of the qualitative data. But upon request, all qualitative and quantitative interview data can be submitted to the journal.

All PLOS journals now require all data underlying the findings described in their manuscript to be freely available to other researchers, either a. In a public repository, b. Within the manuscript itself, or c. Uploaded as supplementary information.

This policy applies to all data except where public deposition would breach compliance with the protocol approved by your research ethics board. If your data cannot be made publicly available for ethical or legal reasons (e.g., public availability would compromise patient privacy), please explain your reasons on resubmission and your exemption request will be escalated for approval.

4. When completing the data availability statement of the submission form, you indicated that you will make your data available on acceptance. We strongly recommend all authors decide on a data sharing plan before acceptance, as the process can be lengthy and hold up publication timelines. Please note that, though access restrictions are acceptable now, your entire data will need to be made freely accessible if your manuscript is accepted for publication. This policy applies to all data except where public deposition would breach compliance with the protocol approved by your research ethics board. If you are unable to adhere to our open data policy, please kindly revise your statement to explain your reasoning and we will seek the editor's input on an exemption. Please be assured that, once you have provided your new statement, the assessment of your exemption will not hold up the peer review process.

5. Please amend the manuscript submission data (via Edit Submission) to include author Melek Güler

6. Please amend your authorship list in your manuscript file to include author Melek KURŞUNEL

7. Please remove your figures from within your manuscript file, leaving only the individual TIFF/EPS image files, uploaded separately. These will be automatically included in the reviewers’ PDF.

8. Please ensure that you refer to Figures 1 and 2 in your text as, if accepted, production will need this reference to link the reader to the figures.

9. We note you have included tables to which you do not refer in the text of your manuscript. Please ensure that you refer to Tables 1, 2, and 5 in your text; if accepted, production will need this reference to link the reader to the Tables.

10. If the reviewer comments include a recommendation to cite specific previously published works, please review and evaluate these publications to determine whether they are relevant and should be cited. There is no requirement to cite these works unless the editor has indicated otherwise.

11. Please review your reference list to ensure that it is complete and correct. If you have cited papers that have been retracted, please include the rationale for doing so in the manuscript text, or remove these references and replace them with relevant current references. Any changes to the reference list should be mentioned in the rebuttal letter that accompanies your revised manuscript. If you need to cite a retracted article, indicate the article’s retracted status in the References list and also include a citation and full reference for the retraction notice.

[Note: HTML markup is below. Please do not edit.]

Reviewers' comments:

Reviewer's Responses to Questions

**Comments to the Author**

1. Is the manuscript technically sound, and do the data support the conclusions?

The manuscript must describe a technically sound piece of scientific research with data that supports the conclusions. Experiments must have been conducted rigorously, with appropriate controls, replication, and sample sizes. The conclusions must be drawn appropriately based on the data presented.

Reviewer #1: Yes

Reviewer #2: Yes

**********

2. Has the statistical analysis been performed appropriately and rigorously?

Reviewer #1: Yes

Reviewer #2: Yes

**********

3. Have the authors made all data underlying the findings in their manuscript fully available?

The PLOS Data policy requires authors to make all data underlying the findings described in their manuscript fully available without restriction, with rare exception (please refer to the Data Availability Statement in the manuscript PDF file). The data should be provided as part of the manuscript or its supporting information, or deposited to a public repository. For example, in addition to summary statistics, the data points behind means, medians and variance measures should be available. If there are restrictions on publicly sharing data—e.g. participant privacy or use of data from a third party—those must be specified.

Reviewer #1: Yes

Reviewer #2: Yes

**********

4. Is the manuscript presented in an intelligible fashion and written in standard English?

PLOS ONE does not copyedit accepted manuscripts, so the language in submitted articles must be clear, correct, and unambiguous. Any typographical or grammatical errors should be corrected at revision, so please note any specific errors here.

Reviewer #1: Yes

Reviewer #2: Yes

**********

5. Review Comments to the Author

Please use the space provided to explain your answers to the questions above. You may also include additional comments for the author, including concerns about dual publication, research ethics, or publication ethics. (Please upload your review as an attachment if it exceeds 20,000 characters)

Reviewer #1: I would like to commend the authors for addressing an important and socially relevant topic. The manuscript entitled “Is having a child with special needs a barrier to physical activity? A mixed design study” investigates a critical issue concerning the health and well-being of parents caring for children with special needs. The topic holds substantial relevance not only for the fields of sport science and physical activity, but also for public health, psychology, disability studies, and family welfare research. By exploring how caregiving responsibilities may influence parents’ physical activity levels, the study provides insights that could contribute to designing more inclusive health promotion strategies and social support systems.

In particular, the use of a mixed-methods approach offers a valuable perspective by combining quantitative findings with qualitative insights. This approach allows the authors to capture both measurable patterns and the lived experiences of parents caring for children with special needs. Such integration has the potential to enrich the literature and inform future interventions targeting parental well-being and physical activity participation.

Overall, the manuscript presents a meaningful contribution to the field and addresses an underexplored research area. However, several minor revisions are needed to strengthen the clarity and academic rigor of the paper. These revisions mainly concern referencing, literature contextualization, and methodological justification, as outlined below.

1. Missing references in the introduction

Some statements in the Introduction section require supporting references. In particular, the following claims should be supported by appropriate scholarly citations:

“The caregiving burden experienced by parents can negatively impact their own and their children's daily living habits. Parents tend to neglect their own health in order to care for their children. It is well-known that parents' physical activity levels do not reach the recommended levels, particularly during the time remaining after caring for children during the day.”

These statements represent important claims about parental health behaviors and caregiving burden. Therefore, they should be supported by relevant empirical studies or systematic reviews to strengthen the academic foundation of the argument. Incorporating recent literature from fields such as caregiving research, parental health behavior, and disability studies would improve the credibility and contextual grounding of the manuscript.

2. Strengthening the literature gap

The manuscript briefly mentions that there is limited research on physical activity barriers among parents of children with special needs. However, the discussion of the research gap could be strengthened.

The authors are encouraged to: 1) present more previous studies examining barriers experienced by parents of children with special needs, particularly in relation to caregiving burden and lifestyle limitations; 2) clarify that while studies may exist regarding caregiver burden, psychological stress, or parenting challenges, few specifically examine physical activity participation among parents; 3) explicitly demonstrate how the current study contributes to filling this gap; and 4) provide a more systematic overview of prior studies will help readers better understand the novelty and significance of the present research.

3. Justification of the mixed-methods approach

The manuscript states that the study employs a mixed-methods design, combining quantitative and qualitative approaches. While the design is appropriate for the research problem, the reasoning for choosing this methodological approach could be elaborated further. Specifically, the authors could strengthen this section by: 1) explaining why quantitative data alone may not sufficiently capture the complexity of parental experiences; 2) clarifying how qualitative interviews help interpret or explain the quantitative findings, particularly regarding perceived barriers; 3) providing a clearer description of the integration of quantitative and qualitative findings in the explanatory sequential design; and 4) expanding this justification will help readers better understand the methodological rationale and enhance the overall methodological transparency of the study.

4. Overall recommendation

The manuscript addresses a significant and meaningful research topic with potential societal impact, particularly in improving awareness of the challenges faced by parents of children with special needs and the implications for their health and well-being. The study design and findings are promising, and the mixed-methods approach offers valuable insights into both measurable trends and lived experiences.

With the minor revisions suggested above—primarily strengthening references, clarifying the research gap, and elaborating the methodological rationale—the manuscript will be considerably improved and better positioned to contribute to the literature.

Reviewer #2: The sequential mixed-methods research design should be cited, for example (Creswell, etc) to acknowledge previous scholars. Also, the thematic analyses ought to be cited and attributed to current acknowledgements based on previous contributions of these analytical framework. A stronger foundation on the theoretical framework would be an added strength to this paper.

**********

6. PLOS authors have the option to publish the peer review history of their article (what does this mean?). If published, this will include your full peer review and any attached files.

If you choose “no”, your identity will remain anonymous but your review may still be made public.

**Do you want your identity to be public for this peer review?**  For information about this choice, including consent withdrawal, please see our Privacy Policy.

Reviewer #1: No

Reviewer #2: No

**********

[NOTE: If reviewer comments were submitted as an attachment file, they will be attached to this email and accessible via the submission site. Please log into your account, locate the manuscript record, and check for the action link "View Attachments". If this link does not appear, there are no attachment files.]

To ensure your figures meet our technical requirements, please review our figure guidelines: https://journals.plos.org/plosone/s/figures

You may also use PLOS’s free figure tool, NAAS, to help you prepare publication quality figures: https://journals.plos.org/plosone/s/figures#loc-tools-for-figure-preparation.

NAAS will assess whether your figures meet our technical requirements by comparing each figure against our figure specifications.

# Author comment: Author response to Decision Letter 1

**DOI:** 10.1371/journal.pone.0348527.r002

14 Apr 2026

Dear Editor: The researchers' names have been updated. Due to her divorce, Melek GÜLER's surname has been changed to Melek KURŞUNEL. Her email address has also been updated. The names of the other researchers have not changed (lines: 8 and 35). Information regarding ethical approval decisions has been added to the Methods section (lines: 245–249). Tables 1, 2 and 5 are referenced in the text (lines: 160, 255 and 295). Figures 2 and 3 are referenced in the text (lines: 273 and 29).

Dear Reviewer 1: Reference sources have been added to support some of the statements in the introduction (lines: 80–83). The final paragraph of the introduction explains why this research was necessary and discusses the research gap in the literature. References have also been added (lines: 112–122). The rationale for using a mixed-methods design is explained in detail in the Methods section (lines: 136–144). The following sources have been added to the references section.( lines: 510, 513, 516, 538 and 542).

Dear Reviewer 2: When discussing mixed methods in research design, the work of Creswell and Clark (2007) is referenced. However, a research design diagram has been added to improve understanding of the design (line: 146).

Attachment Submitted filename: Response to Reviewers.docx

# Peer review: Decision Letter 1

**DOI:** 10.1371/journal.pone.0348527.r003

**Authors:** Hidetaka Hamasaki, Hidetaka Hamasaki

2 Jun 2026

Is having a child with special needs a barrier to physical activity? A mixed design study

PONE-D-25-53816R1

Dear Dr. KURŞUNEL,

We’re pleased to inform you that your manuscript has been judged scientifically suitable for publication and will be formally accepted for publication once it meets all outstanding technical requirements.

Within one week, you’ll receive an e-mail detailing the required amendments. When these have been addressed, you’ll receive a formal acceptance letter and your manuscript will be scheduled for publication.

An invoice will be generated when your article is formally accepted. Please note, if your institution has a publishing partnership with PLOS and your article meets the relevant criteria, all or part of your publication costs will be covered. Please make sure your user information is up-to-date by logging into Editorial Manager at Editorial Manager® and clicking the ‘Update My Information' link at the top of the page. For questions related to billing, please contact billing support.

If your institution or institutions have a press office, please notify them about your upcoming paper to help maximize its impact. If they’ll be preparing press materials, please inform our press team as soon as possible -- no later than 48 hours after receiving the formal acceptance. Your manuscript will remain under strict press embargo until 2 pm Eastern Time on the date of publication. For more information, please contact onepress@plos.org.

Kind regards,

Hidetaka Hamasaki

Academic Editor

PLOS One

Additional Editor Comments (optional):

Reviewers' comments:

Reviewer's Responses to Questions

**Comments to the Author**

1. If the authors have adequately addressed your comments raised in a previous round of review and you feel that this manuscript is now acceptable for publication, you may indicate that here to bypass the “Comments to the Author” section, enter your conflict of interest statement in the “Confidential to Editor” section, and submit your "Accept" recommendation.

Reviewer #2: All comments have been addressed

**********

2. Is the manuscript technically sound, and do the data support the conclusions?

The manuscript must describe a technically sound piece of scientific research with data that supports the conclusions. Experiments must have been conducted rigorously, with appropriate controls, replication, and sample sizes. The conclusions must be drawn appropriately based on the data presented.

Reviewer #2: Yes

**********

3. Has the statistical analysis been performed appropriately and rigorously?

Reviewer #2: Yes

**********

4. Have the authors made all data underlying the findings in their manuscript fully available?

The PLOS Data policy requires authors to make all data underlying the findings described in their manuscript fully available without restriction, with rare exception (please refer to the Data Availability Statement in the manuscript PDF file). The data should be provided as part of the manuscript or its supporting information, or deposited to a public repository. For example, in addition to summary statistics, the data points behind means, medians and variance measures should be available. If there are restrictions on publicly sharing data—e.g. participant privacy or use of data from a third party—those must be specified.

Reviewer #2: Yes

**********

5. Is the manuscript presented in an intelligible fashion and written in standard English?

PLOS ONE does not copyedit accepted manuscripts, so the language in submitted articles must be clear, correct, and unambiguous. Any typographical or grammatical errors should be corrected at revision, so please note any specific errors here.

Reviewer #2: Yes

**********

6. Review Comments to the Author

Please use the space provided to explain your answers to the questions above. You may also include additional comments for the author, including concerns about dual publication, research ethics, or publication ethics. (Please upload your review as an attachment if it exceeds 20,000 characters)

Reviewer #2: a sound and convincing revised version of previous manuscript. the mixed design approach provided an in-depth and displayed a rigorous attempt to delved into the psyche of parental perceptions and behavior towards care of special needs chilren

**********

7. PLOS authors have the option to publish the peer review history of their article (what does this mean?). If published, this will include your full peer review and any attached files.

If you choose “no”, your identity will remain anonymous but your review may still be made public.

**Do you want your identity to be public for this peer review?**  For information about this choice, including consent withdrawal, please see our Privacy Policy.

Reviewer #2: No

**********

# Editor report: Acceptance letter

**DOI:** 10.1371/journal.pone.0348527.r004

**Authors:** Hidetaka Hamasaki, Hidetaka Hamasaki

PONE-D-25-53816R1

PLOS One

Dear Dr. KURŞUNEL,

I'm pleased to inform you that your manuscript has been deemed suitable for publication in PLOS One. Congratulations! Your manuscript is now being handed over to our production team.

At this stage, our production department will prepare your paper for publication. This includes ensuring the following:

* All references, tables, and figures are properly cited

* All relevant supporting information is included in the manuscript submission,

* There are no issues that prevent the paper from being properly typeset

You will receive further instructions from the production team, including instructions on how to review your proof when it is ready. Please keep in mind that we are working through a large volume of accepted articles, so please give us a few days to review your paper and let you know the next and final steps.

Lastly, if your institution or institutions have a press office, please let them know about your upcoming paper now to help maximize its impact. If they'll be preparing press materials, please inform our press team within the next 48 hours. Your manuscript will remain under strict press embargo until 2 pm Eastern Time on the date of publication. For more information, please contact onepress@plos.org.

You will receive an invoice from PLOS for your publication fee after your manuscript has reached the completed accept phase. If you receive an email requesting payment before acceptance or for any other service, this may be a phishing scheme. Learn how to identify phishing emails and protect your accounts at https://explore.plos.org/phishing.

If we can help with anything else, please email us at customercare@plos.org.

Thank you for submitting your work to PLOS ONE and supporting open access.

Kind regards,

PLOS ONE Editorial Office Staff

on behalf of

Dr. Hidetaka Hamasaki

Academic Editor

PLOS One
